# *Notes from the Field:* Vibriosis Cases Associated with Flood Waters During and After Hurricane Ian — Florida, September–October 2022

**DOI:** 10.15585/mmwr.mm7218a5

**Published:** 2023-05-05

**Authors:** Nicole Sodders, Kimberly Stockdale, Kayla Baker, Arielle Ghanem, Benjamin Vieth, Terri Harder

**Affiliations:** ^1^Florida Department of Health; ^2^Florida Department of Health in Lee County, Fort Myers, Florida; ^3^Florida Department of Health in Collier County, Naples, Florida.

On the afternoon of September 28, 2022 (epidemiologic week 40), Hurricane Ian made landfall on Florida’s southwest coast as a category 4 hurricane with maximum sustained winds of 150 miles per hr (241 km per hr). The storm surge (the abnormal rise in seawater related to a storm) reached 12–18 ft (3.6–5.5 m) above ground level in some coastal areas of Lee and Collier counties, on southwest Florida’s Gulf coast (*1*). In the days after the hurricane, a notable increase in cases of vibriosis was observed by the Florida Department of Health: 38 cases and 11 vibriosis-associated deaths were attributed to the storm. During the hurricane response, Florida deployed public health messaging on storm preparedness statewide, advising residents of 1) the importance of not wading in flood waters or in standing water after a storm, especially persons with open wounds; 2) the potential life-threatening illness that can be caused by *Vibrio vulnificus;* and the importance of seeking prompt medical attention if symptoms* are experienced (*2*).

*Vibrio* bacteria thrive in warm salty or brackish waters such as those pushed ashore in a storm surge. These virulent gram-negative bacteria can result in gastrointestinal illness after consumption of raw or undercooked shellfish, or a skin infection following exposure of an open wound to salt or brackish water (a mixture of freshwater and seawater) (*3*). This data was obtained through routine investigation of vibriosis cases and follow- up using Florida’s reportable disease surveillance system Merlin. This activity was reviewed by CDC and was conducted consistent with applicable federal law and CDC policy.^†^

In the week preceding the hurricane (week 39), southwest Florida reported no vibriosis cases, which was below the 5-year median (three cases) for that week. Based on epidemiology from previous years, three vibriosis cases were expected to be reported in southwest Florida in the weeks during and after the storm (weeks 40 and 41). However, 38 culture-confirmed vibriosis cases determined to be associated with the impacts of Hurricane Ian occurred in Lee and Collier counties during September 29–October 23, representing an 1,100% increase over the 5-year median. These cases included 29 (76%) caused by *V. vulnificus*, three cases (8%) by *Vibrio cholerae* type non-O1, and two cases (5%) each by *Vibrio parahaemolyticus*, *Vibrio* *fluvialis*, and other *Vibrio* spp. One case occurred in a resident of another state who was exposed in Florida, and one patient was co-infected with *V. vulnificus* and *V. parahaemolyticus*. The median patient age was 80 years (range = 51–94 years), and 79% of the 38 cases occurred in men. Date of symptom onset ranged from September 28 to October 9; onset dates for two patients were unknown. Among 36 (95%) patients with known illness onset date, 34 (94%) cases occurred within 6 days of storm landfall (Figure).

**FIGURE:**
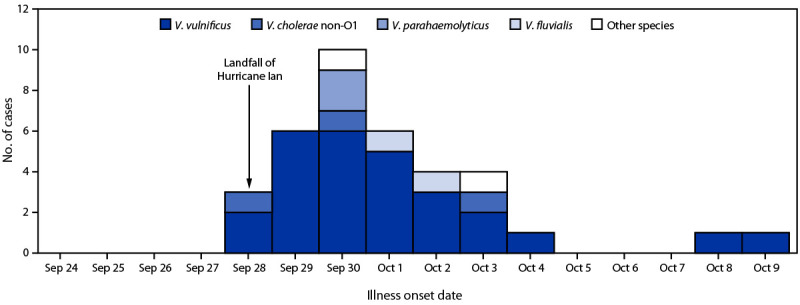
Hurricane Ian–associated vibriosis cases* (N = 38) and deaths^†^ (N = 11), by illness onset date and* Vibrio* species — Florida, September 28–October 9, 2022 * Two cases not displayed because illness onset date was unknown. ^†^ Nine *V. vulnificus*–associated deaths occurred in patients with illness onset dates of September 29 (three), September 30 (four), and October 9 (one); the onset date for one *V. vulnificus*–associated case was not known; one *V. cholerae* non-O1–associated death occurred in a patient with illness onset on September 28, and one death associated with another *Vibrio* sp. occurred in a patient with illness onset on September 30. One *V. vulnificus*–associated death was in a patient coinfected with *V. parahaemolyticus*.

Thirty-three (87%) patients had skin infections associated with exposure to storm surge or flood waters; two (5%) reported wounds with unclear exposures; two (5%) reported prolonged exposure to flood waters after being trapped in their homes or while evacuating; and one patient (3%) reported drinking flood waters. Thirty-six patients were hospitalized for a median of 10 days (range = 1–51 days), and eight (22%) were transferred to skilled nursing facilities or rehabilitation facilities after hospitalization. Eight patients required skin grafts, and three underwent lower extremity amputations. Among the 11 (29%) deaths that occurred, nine occurred in patients infected with *V. vulnificus*, and one death each occurred in patients infected with *V. cholerae* non-O1 and with some other *Vibrio* sp.

This outbreak is notable because of the large number of hurricane-attributable cases that occurred during a short period. The case fatality rate was 28.9%, which might be related to the age of many of the patients. In 2017, after Hurricane Irma, six storm-related vibriosis cases were documented in Florida, approximately one sixth the number documented after Ian, possibly because of the lower storm surge (5–6 ft [1.5–1.8 m]) during Irma. In 2005, Hurricane Katrina resulted in the highest storm surge in U.S. history (>25 ft [7.6 m]); 22 vibriosis cases were linked to that storm, all of which resulted in disease onset within 7 days of storm landfall (*4*). Communicating risk information to the public and to health care providers before an event with potential for significant storm surge, such as a major hurricane, might prevent cases and assist in the timely diagnosis and treatment of vibriosis. In hurricane-associated wound infections, *V. vulnificus* should be considered as a possible cause, aggressive wound care and prompt administration of antibiotics is essential to improving survival (*5*).

## Footnotes

* Signs and symptoms include fever, chills, decreased blood pressure (septic shock), and skin lesions.
